# What is the effect of spinal manipulation on the pressure pain threshold in young, asymptomatic subjects? A randomized placebo-controlled trial, with a cross-over design

**DOI:** 10.1186/s12998-020-0296-1

**Published:** 2020-02-07

**Authors:** Margaux Honoré, Mathieu Picchiottino, Niels Wedderkopp, Charlotte Leboeuf-Yde, Olivier Gagey

**Affiliations:** 1 CIAMS, University of Paris-Sud, University of Paris-Saclay, F-91405 Orsay Cedex, France; 2 CIAMS, University of Orléans, F-45067 Orléans, France; 3 Institut Franco Européen de Chiropraxie, 24 boulevard Paul Vaillant-Couturier, F-94200 Ivry sur Seine, France; 4 Institute for Regional Health Research, University of Southern Denmark, Odense, Denmark; 5 Orthopedic Department, Hospital of Southwestern Jutland, Esbjerg, Denmark

**Keywords:** Spinal manipulation, Pressure pain threshold, Asymptomatic subjects, Randomized controlled trial, Sham, Effect, Duration, Effect size, Manipulation vertébrale, Seuil de douleur de pression, Sujets asymptomatiques, Essai contrôlé randomisé, Placébo, Effet, Durée, Taille de l’effet

## Abstract

**Background:**

Spinal manipulation (SM) has been shown to have an effect on the pressure pain threshold (PPT) in asymptomatic subjects, but SM has never been compared in studies on this topic to a validated sham procedure. We investigated the effect of SM on the PPT when measured i) in the area of intervention and ii) in an area remote from the intervention. In addition, we measured the size and duration of the effect.

**Method:**

In a randomized cross-over trial, 50 asymptomatic chiropractic students had their PPT measured at baseline, immediately after and every 12 min after intervention, over a period of 45 min, comparing values after SM and a previously validated sham. The trial was conducted during two sessions, separated by 48 h. PPT was measured both regionally and remotely from the ‘treated’ thoracic segment. Blinding of study subjects was tested with a post-intervention questionnaire. We used mixed linear regression with the baseline value and time as co-variates. If a significant difference were found between groups, then an effect size would be calculated using Cohen’s d or Hedge’s h coefficient. Statistical significance was set at *p* < 0.05.

**Results:**

Study subjects had been successfully blinded. No statistically significant differences were found between SM and sham estimates, at any time or anatomical location.

**Conclusion:**

When compared to a valid sham procedure and with successfully blinded subjects, there is no regional or remote effect of spinal manipulation of the thoracic spine on the pressure pain threshold in a young pain-free population.

## Resume

**Contexte:**

La manipulation vertébrale (MV) a montré un effet sur le seuil de douleur à la pression (SDP) chez les sujets asymptomatiques, mais la MV n’a jamais été comparée dans les études sur le sujet à une procédure placébo validée. Nous avons étudié l’effet de la MV sur le SDP lorsque mesuré i) dans la zone d’intervention, ii) dans une zone éloignée de l’intervention. De plus, la grandeur et la durée de l’effet ont été évaluées.

**Méthode:**

Un essai randomisé croisé portant sur cinquante jeunes étudiants en chiropratique asymptomatiques. Le SDP a été mesuré au départ, immédiatement après et toutes les douze minutes après l’intervention, sur une période de 45 min, en comparant les valeurs après MV et un placébo valide. L’essai a été conduit en deux sessions, séparées de 48 h. Le SDP a été mesuré à la fois régionalement et à distance du segment thoracique “traité” , sur une période de 45 min. L’aveuglement était testé avec un questionnaire post-intervention. Nous avons utilisé une régression linéaire mixte avec la mesure de référence et la durée comme co-variables. La taille de l’effet a été calculée, à l’aide du coefficient *d* de Cohen ou *h* de Hedge, après constat d’une différence significative entre les groupes. La signification statistique a été fixée à *p* < 0,05.

**Résultats:**

Les sujets de l’étude ont été aveuglés avec succès. Aucune différence statistiquement significative n’a été constatée entre les estimations MV et le placébo, à aucun moment, ni à aucune zone du rachis.

**Conclusion:**

Comparé à une procédure placébo valide et à des sujets aveuglés avec succès, il n’existe aucun effet régional ou à distance de la manipulation vertébrale de la colonne thoracique sur le seuil de douleur à la pression chez une population jeune et indolore.

## Introduction

### Background

Spinal manipulation (SM) can be defined as a high velocity low amplitude forced manoeuvre applied to spinal joints outside the range of motion but within the normal range of anatomical joints, sometimes accompanied by a characteristic cracking sound [1]*.* It can be compared to a mobilisation, which also is defined as a type of manual therapy with a comparable execution and similar clinical results, but applied slower and/or repetitively over the joints within the range of motion and within the patient’s control [2, 3] SM has been shown, sometimes, to have a clinical impact in the treatment of musculoskeletal pain [4], although the mechanisms underlying the reduction of pain are not yet well defined. Such mechanisms can be studied by the means of experimentally induced pain.

#### Spinal manipulation and experimentally induced pain

##### Asymptomatic subjects

Experimentally induced pain can be used both in clinical and pain-free populations. The advantage of using study subjects from the asymptomatic population is that it makes it possible to deal with the ‘normal’ situation, as they are likely to have a normally functioning pain management system; as opposed to people with chronic pain, who are likely to have a dysfunction of the descending pain inhibitory mechanisms [5]. Therefore, studies of asymptomatic and symptomatic subjects may provide different insights on the treatment for pain. Also, changes in pain perception arise late in life [6] with a decrease of pain sensitivity, so purely experimental studies are often performed on young asymptomatic people.

##### Pressure pain

Pain can be induced in many ways in laboratory-controlled conditions, one of the most common being pain induced by pressure. The pressure pain threshold (PPT) is defined as the minimal pressure that provokes pain or discomfort [7]. PPT is commonly used in pain research. As everybody has a pain threshold, regardless pain status, PPT can be used also on pain-free subjects. It is tested using an algometer, which measures the exact pressure applied in a specific location, making it possible to determine the precise threshold.

##### Regional and remote effect of spinal manipulation on the pressure pain threshold

A previous systematic review on studies including asymptomatic subjects [8] showed that SM could significantly and more consistently reduce pain induced by pressure, as opposed to other kinds of induced pain, at least when testing the pain sensation in the same area as the manipulated zone or along the same dermatome (‘regionally’). More precisely, 12/20 studies included in the review showed a positive effect on the PPT when measured regionally, and 5/9 studies reported a remote effect (i.e. outside the manipulated area or its dermatome) on experimentally induced pain [8]. Unfortunately, none had a blind assessor, making it impossible to completely trust the results. More knowledge is thus needed concerning both the regional and remote effect of spinal manipulation.

#### Spinal manipulation compared to a sham procedure

##### “Credible” sham procedure

In research on the effect of manual therapy, the question of a “credible” sham procedure is challenging, as study subjects can easily deduce if they are treated or not. A recent systematic review [9] on the effect of SM on the PPT in the area corresponding to the SM (i.e. ‘regionally’) included an assessment of the credibility of the sham procedures in eight randomized controlled trials, by taking into account the psychological part of the placebo (i.e. can the subjects spot the difference between the “real” intervention or the placebo?) but also the physiological part of it (i.e. do the physical aspects of the sham procedure resemble the “real” intervention?). A completely “credible” placebo would fulfil both criteria. It was found that, in these studies when compared with a reasonably “credible” placebo procedure, a positive regional effect of SM was found as measured on the PPT. Surprisingly, no significant regional effect was reported when the placebo procedure was not considered credible at all. The credibility of the sham could be an important factor to consider when dealing with manual therapies studies, because the credibility of the placebo seems to affect the results.

Another factor to consider with sham interventions is the need to use a “thrust” to mimic the “real” intervention as much as possible, which means that it must be delivered outside the range of spinal joints. A recent study assessed the credibility of such a sham with a lateral and light “thrust” on the scapulae rather than on the thoracic joints. It was validated immediately after each of 12 treatment sessions over 3 months by post-treatment questionnaires with more than 80% of success (i.e. more than 80% of the subjects did not spot the difference between a “real” intervention and the sham intervention) [10]. Previously, it has been unusual that researchers check whether the sham intervention was recognized as such, or whether study subjects were ‘fooled’ by the procedure. Therefore, the effect of SM on PPT in asymptomatic subjects should be challenged by a validated sham procedure**.**

#### Size and duration of the effect of spinal manipulation

Previous analyses of eight randomized controlled trials that had investigated the regional effect of SM on the PPT in asymptomatic subjects showed the effect size to be ‘*medium’ (Cohen’s d*: *0.2–0.5)* immediately after the intervention. Five minutes after intervention, the effect size was ‘*mainly large*’ (Cohen’s d ≥ 0.8), and it was ‘*mainly medium*’ 10 min after intervention [11]. Therefore, it was concluded that the effect was probably rather short-lasting but should be investigated for a longer period, as the included studies did not continue their measurements further than 30 min. Clearly, more information is needed on the element of time.

In conclusion, more knowledge is needed concerning the regional and remote effects of spinal manipulation, when compared to a valid sham procedure, as well as we need to know more about its duration and size, starting with asymptomatic subjects. For these reasons, we conducted a study on asymptomatic subjects to obtain answers to the following questions:

What is the effect of spinal manipulation on the pressure pain threshold when being compared with a valid sham procedure when measured i) in the area of intervention and ii) in an area remote from the intervention? If there is an effect, what is its i) size and ii) duration?

## Method

### Design, ethics committee and registration

This study is a randomized sham-controlled trial with a cross-over design. The experiment took place in a research laboratory at the Institut Franco-Européen de Chiropraxie in Ivry sur Seine, France, from September 2017 to October 2018, with breaks over the school holidays. This report deals with the second part of a larger study, in which data were collected to investigate the effect of SM on both i) the autonomic system and ii) pain perception in asymptomatic individuals. Thus, the present report deals only with information relevant to the pain perception study. For more details, please see Picchiottino et al., 2019 [12]. The study was approved by the ethics committee EA 4532 of the University Paris Sud UFR STAPS, Orsay, France (October, 2016), registered as a clinical trial at https://clinicaltrials.gov (Registration NCT03776708), and, as required by French law, insured by HDI global assurance (N°01012787–14,009).

### Study protocol

**Fig. 1:**
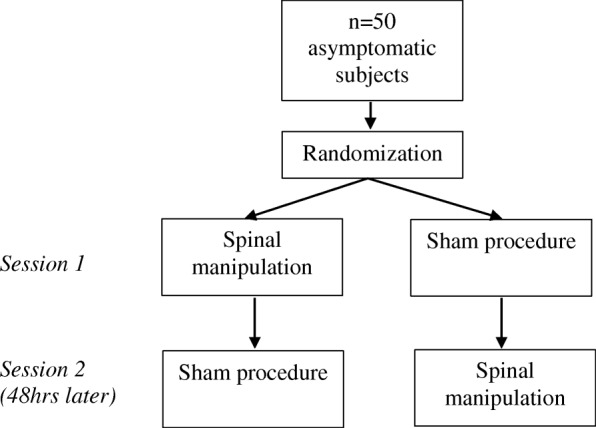
Randomized sham-controlled study with a cross-over design, taking place in two sessions separated by 48 h

The experiments were carried out during two separate sessions. In the first session, subjects were randomly divided in two groups to receive either SM or a placebo procedure (‘sham’) by choosing a sealed and non-transparent envelope in an opaque box. They would thus choose the order of interventions at the first visit. The second session took place 48 h later, when the subjects received the second type of intervention, SM or placebo, the opposite to what they received the first time (Fig. 1). The second session was scheduled at the same time of the day as the first session, the duration of each being about an hour.

**Fig. 2:**
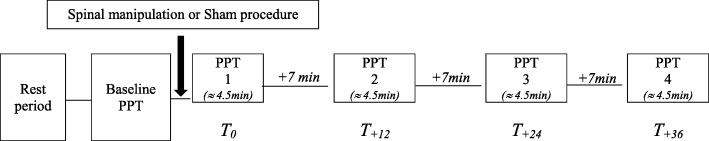
Pressure pain threshold data collection at baseline and every twelve minutes post-intervention in the thoracic and lumbar spine. Pressure pain threshold (PPT); PPT 1 is collected immediately (T0 up to T + 4.5 min) after intervention, PPT 2 is collected twelve minutes (T + 12 up to T + 16.5 min) after intervention, PPT 3 is collected twenty-four minutes (T + 24 up to T + 28.5 min) after intervention, and PPT 4 is collected thirty-six minutes (T + 36 up to T + 40.5 min) after intervention

Each session started with a short period of rest. This was followed by the PPT measurements recordings before any intervention, both in the thoracic and in the lumbar spine, after which either SM or a sham intervention took place. These PPT measurements were repeated every 12 min, i.e. in total four times after each intervention over a period of 45 min (Fig. 2).

The experiment took place in a room with a stable and comfortable temperature with subjects lying prone on a treatment table. A ‘fake’ measurement of the PPT was done on the subjects with an algometer before the beginning of the study to initiate them to the procedure and to prevent anxiety.

A licensed chiropractor performed the interventions. The PPT assessor was trained for more than an hour in the use of the algometer, as previously recommended [13]. This person was blinded to the group allocation of study subjects. The study subjects could not see their own PPT readings, to prevent them from consciously affecting their values. They were instructed not to communicate with the investigator about the intervention they received.

The collected data were used to see if the PPT increased more after SM than after a sham intervention.

Study subjects consisted of first-year chiropractic students, who theoretically could be biased. as they were not naïve to SM. This required careful considerations. The procedures relating to them will therefore be explained in detail below.

### Recruitment

With the permission of the College, posters and presentations were distributed at the beginning of the academic year to spark an interest in the students for research projects. Students potentially interested in the study were invited to come to the research laboratory, where an information letter was given to them, including the informed consent sheet they would have to sign to participate. They were explained the potential risks of the interventions and their rights to withdraw at any moment. All information linking the study subjects to their data was protected during the experiment and destroyed at the end of the study. No individual student could therefore be identified in the data file after the experiment nor in the final report.

### Inclusion and non-inclusion criteria

Included in the study were consenting, asymptomatic students between 18 and 40 years old. ‘Asymptomatic’ was defined as having no pain in the tested area. Further, they should not report having had any spinal pain lasting more than a month, having taken painkillers 24 h prior to the study, or having received manual treatment during the previous 48 h. There should be no contra-indications to spinal manipulation, such as instability (fracture, malformation), bony or ligamentous fragility, or local inflammation. Informed consent was obtained from potential study subjects by the treating chiropractor.

The data collection also included autonomic nervous system variables [12], so the students were informed not to ingest food, caffeine, alcohol, or tobacco in the hour prior to the sessions, as well as not to perform intense physical activity the day of the experimentation.

### Spinal manipulation and sham procedure

The SM was performed on all participants at the level of the fifth thoracic vertebra (with a margin of palpatory error), making sure this was a pain free area on light palpation. The maneuver was of high velocity with low amplitude, oriented posterior to anterior, with the contact hand placed over the transvers process area of the c vertebra.

In order to imitate the SM to a maximum, the sham procedure consisted of a manual contact on the right medial angle of the scapula with both hands. After putting the tissues briefly under tension, a slight movement with a thrust was performed, respecting the scapula-thoracic sliding planes laterally without influencing the spine. It was considered by us to be a “credible” sham procedure, as it resembles an actual act of thoracic manipulation being performed over the back and included manual contacts and movements used in manual therapy but lacked the precise action over a spinal joint. We selected this method to confuse the study subjects, who had been told that they would be subjected to different manipulative techniques. An almost identical version of this method has been previously validated with good results [10].

### Blinding

The study subjects were first year chiropractic students. Throughout their undergraduate studies, these students will be exposed to several types of spinal manipulations. However, in their first year of study they would be unlikely to have enough knowledge and experience of manipulation to know about the various types that exist and, therefore, unlikely to discover a well performed sham intervention, if it was not directly contrasted to manipulation (i.e. manipulation vs. sham).

These subjects were therefore told that the aim of the study was to assess the outcomes of different efficacious techniques used in manual therapies, and that they would receive the same type of intervention during both sessions. During the random allocation procedure, they had the choice between six envelopes (to reinforce the idea that there were many intervention possibilities). They were also not informed of their treatment allocation throughout the study.

With this procedure, we made sure they were naïve to the exact objectives of our study with the intent to blind them to the type of intervention.

Further, the study subjects responded to a questionnaire at the end of each session to see what their beliefs regarding the effectiveness of each intervention was (see Additional file 1). A completely blinded subject would have the same beliefs for both interventions, and might even suppose the sham more effective than the real SM. As reported later, the results showed that they had been successfully blinded.

### Algometer

An algometer type 2 (*SOMEDIC Electronics, Sweden*) was used to take these measurements This algometer has a circular metal tip of 1cm^2^, which is to be applied perpendicularly to the skin with a normalized speed (the pressure exerted is 50 kPa/s). Subjects were instructed to press a switch, when they felt the gradual pressure turning into pain. The reading was then frozen at this level, as indicated on the screen of the algometer, then transferred manually onto paper and then entered in Excel software. Data were entered separately and blindly by two people and then checked for accuracy. These data were stored, unchangeable and confidential.

### Measurements

The algometer is reported to have good reliability [14, 15], sensitivity [15] and specificity [15], which makes it a simple and efficient tool to use, after training the assessor [15]. It was calibrated frequently during data collection to ensure precise values of the PPT. We performed three PPT readings at 30 s interval, at each time of recording, as recommended [16] and at each site. The brief pause between readings is necessary to avoid sensitization of the skin. A cut-off pressure value of 1000 kPa was set for safety purposes [17].

### Statistical analysis

#### Preliminary analyses

Data were analyzed with Stata (version 15.1) software. We ensured the blindness of the statistician by de-identifying the intervention groups (called A and B). Sample size calculation was performed using a repeated measures approach. We found that we would need 43 subjects in each group to show at least a difference of 15% of change between groups (‘supposed’ percentage of clinical significance [18]), with the mean pressure pain threshold of 500 kg/cm2 at baseline. However, as this minimum clinical difference of the PPT is not clearly defined [18], the basis for this power calculation was purely speculative.

Descriptive data were presented as means and standard deviation for each group, at baseline and at the subsequent follow-up times. The distribution of the data was assessed visually with histograms and boxplots. A secondary analysis, where sex was included as covariate, was performed using mixed regression, and we tested for period-group interaction, with *p* = 0.1.

Subcutaneous fat could affect pain produced by pressure but there were no obese people in this study **(**Additional file 2**).** Therefore, BMI was not included in the analysis, although it might well be relevant in other study populations.

Age could influence pain perception, but the range in our study subjects was too narrow to be of any importance. We did not include any psychological variables such as fear avoidance, as we did not think this likely to have an influence on purely experimental pain without any previous suffering or secondary effect on life-style and psychological profile.

#### Testing the effect

We used linear mixed models with random intercept to estimate the adjusted difference in PPT between SMT and sham at each follow-up time point. A separate repeated-measures model was created for the regional and remote PPT outcomes. The dependent variable was the mean of three PPT measurements taken at each time point, the independent variable was intervention (SMT/sham), and the covariates were the session baseline and time. If a significant difference were found between groups, then an effect size would be calculated using Cohen’s d or Hedge’s h coefficient [11]. Statistical significance was set at *p* < 0.05.

## Results

### Descriptive analysis of data

Fifty-one study subjects (male *n* = 23 and females *n* = 28; mean age 20 (+/− 3); range 18–37, (with one subject aged 37) were recruited and randomly assigned into a spinal manipulation group (*n* = 26 in the first session, i.e. equal allocation) or a sham procedure group for the first session. Thus, study subjects served as their own controls, switching to the other type of intervention at the second session.

Data were removed from the final analysis for one study subject because of malfunction of the algometer, resulting in 50 participants for the analyses of the regional PPT tests. For the remote PPT testing, another four subjects were excluded: one, because the lumbar region was painful at the second session and three because they had PPT values over 1000 kPa.

The post-trial questionnaires showed that 78% of the subjects had the same beliefs for both interventions or (although rarely) thought the sham superior to SM, suggesting a successful blinding (Table 1, rows A, B, C). The distributions were found to be within the ‘normal’ ranges both for the regional and remote values. No period-group interaction was found (*p* > 0.25).

*1/ Is there a regional effect of spinal manipulation on the pressure pain threshold when compared to a valid sham in asymptomatic subjects over time?*

**Table 1:** Expectation questionnaire of the subjects between both sessions of the experiment

	Beliefs regarding SM or sham	Total of subjects (n/50)	Percentage (%)	Are the subjects considered completely blinded? (Yes/No)
A	Subjects had same beliefs for SM and sham	32/50	64%	Yes (78%)
B	Subjects thought that both interventions were effective and Sham > SM	1/50	2%	
C	Subjects did not know if SM was effective but thought that the Sham was effective	6/50	12%	
D	Subjects thought that both interventions were effective and SM > Sham	2/50	4%	No (22%)
E	Subjects did not know if the sham was effective but thought that SM was effective	3/50	6%	
F	Subjects did not know if the SM was effective but thought that the Sham was ineffective	3/50	6%	
G	Subjects thought that SM was effective and Sham ineffective	3/50	6%	

-Sham > SM means stronger certainty for the Sham

-SM > Sham means stronger certainty for the SM

*SM* spinal manipulation

The adjusted differences in PPT readings between SM and the sham procedure at regional and remote sites over time are shown in Table 2. The estimates were similar between groups and no statistically significant differences were found between groups at any of the follow-up times (*p* > 0.05).

*2/ Is there a remote effect of spinal manipulation on the pressure pain threshold when compared to a valid sham in asymptomatic subjects over time?*

**Table 2:** Adjusted differences in pressure pain threshold (PPT) readings in kPa at regional testing site at four different times after the interventions

Time measurements	SPINAL MANIPULATION			SHAM procedure			Differences between groups (95% CI)	*P* value
	Means (SD)	Min-Max Values	95% CI	Means (SD)	Min-Max values	95% CI		
Baseline	406 (158)	118–873	361–451	416 (162)	143–877	370–462	−10 (−73;53)	0.76
PPT 1	446 (169)	125–1048	395–487	447 (154)	173–768	389–482	−1 (−64;63)	0.98
PPT 2	450 (168)	137–903	401–494	438 (159)	145–807	386–478	12(−53;77)	0.72
PPT 3	443 (158)	125–853	394–487	438 (180)	151–962	387–480	4(−50;58)	0.90
PPT 4	444 (161)	146–823	395–487	432 (167)	136–859	378–470	12(−51;75)	0.71

*SD* standard deviation, *SE* standard error, *PPT* pressure pain threshold, *PPT 1* follow-up time 1 immediately after intervention, *PPT2* follow-up time 2 at T + 12 min after intervention, *PPT 3* follow-up time 3 at T + 24 min after intervention, *PPT 4* follow-up time 4 at T + 36 min after intervention

The adjusted differences in PPT readings between SM and sham procedure at regional and remote sites over time are shown in Table 3**.** There were somewhat larger differences than for the regional estimates, but no statistically significant differences were found between groups at any of the follow-up times (p > 0.05).

**Table 3:** Adjusted differences in pressure pain threshold (PPT) readings in kPa at remote testing site at four different times after the interventions

Time measurements	SPINAL MANIPULATION			SHAM procedure			Differences between groups (95% CI)	*P* value
	Means (SD)	Min-Max values	95% CI	Means (SD)	Min-Max values	95% CI		
Baseline	511 (166)	221–894	462–568	479 (154)	187–831	432–525	32(− 33;97)	0.34
PPT 1	564 (192)	248–1027	517–623	536 (172)	203–916	485–588	27(− 12;66)	0.47
PPT 2	576 (199)	205–1128	529–635	523 (176)	190–950	471–576	53(− 10;96)	0.18
PPT 3	579 (204)	190–1097	533–639	533 (177)	170–910	486–581	38(−48;124)	0.23
PPT 4	573 (203)	207–1195	527–633	524 (160)	197–817	480–570	49(− 53;151)	0.19

*SD* standard deviation, *SE* standard error, *PPT* pressure pain threshold, *PPT 1* follow-up time 1 immediately after intervention, *PPT2* follow-up time 2 at T + 12 min after intervention, *PPT 3* follow-up time 3 at T + 24 min after intervention, *PPT 4* follow-up time 4 at T + 36 min after intervention

**Fig. 3:**
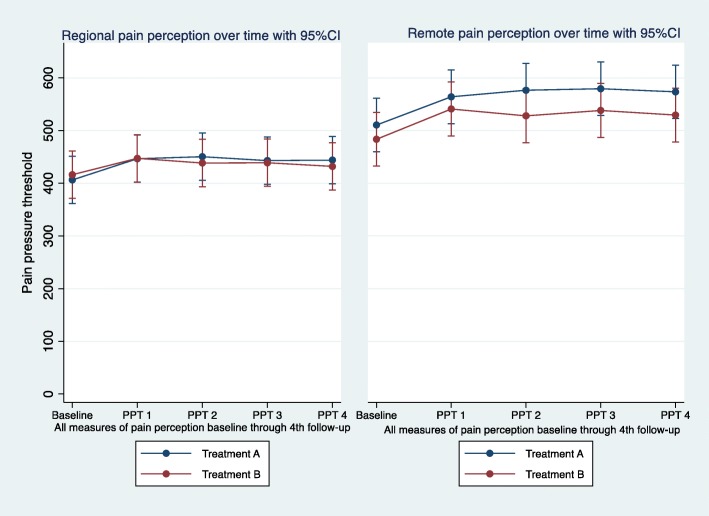
Changes in pressure pain thresholds (PPT) for regional and remote in pain perception after spinal manipulation (treatment A) and sham (treatment B) predicted from mixed linear regression, at baseline and at each follow-up. PPT 1 is collected immediately (T0 up to T + 4.5 min) after intervention, PPT 2 is collected twelve minutes (T + 12 up to T + 16.5 min) after intervention, PPT 3 is collected twenty-four minutes (T + 24 up to T + 28.5 min) after intervention, and PPT 4 is collected thirty-six minutes (T + 36 up to T + 40.5 min) after intervention. Treatment A: spinal manipulation / Treatment B: sham procedure

The changes over time in the pressure pain threshold after the interventions in both regional and remote testing have been visualized in Fig. 3.

*3/ If there is an effect, what is the duration of the regional and remote effect of spinal manipulation on the pressure pain threshold in asymptomatic subjects?*

No statistically significant differences were found between the interventions over time at any of the testing sites. Hence, no duration of effect can be reported.

*4/ If there is an effect, what is the effect size of spinal manipulation on the pressure pain threshold in asymptomatic subjects over time at both regional and remote testing sites?*

As there are no regional or remote effects reported, the effect size is irrelevant.

## Discussion

### Summary of findings

Although several studies have been conducted on the subject, this is the first experimental study testing the effect of spinal manipulation on pressure pain threshold in asymptomatic subjects using a sham, which was shown to be valid with a post-intervention questionnaire. No effect was found for the pressure pain threshold in the area of the intervention (thoracic spine), nor in an area further away (lumbar spine) immediately after SM. Additional measurements were taken over 45 min, which did not change the results.

### Comparison with literature

Our findings are in contradiction with a recent review on the same subject (the effect of SM in asymptomatic regions), which identified 19 studies measuring the PPT [8]. Only 13 of these compared SM with a sham procedure, of which 11 reported a positive effect. Two studies, similarly to us, tested thoracic SM; one with a positive effect. Although the authors of this review examined the general quality of the studies, they did not investigate the validity of the sham.

A second review, also with a positive conclusion in relation to SM in pain free study subjects, further investigated the quality of the sham [9] and found a positive effect in 5 of 8 sham-controlled studies. None of these sham interventions were validated after intervention. A major difference with other studies was, therefore, that our trial used a previously validated sham procedure that imitated a proper SM in all aspects expect in the area and direction of the thrust, as it was performed over the scapula in the plane of the thoracic cage [10]. Very importantly, we also confirmed with a post-intervention questionnaire, if study subjects had identified the ‘effective’ intervention from the ‘ineffective’ intervention, which they had not. These results are strengthened by similar findings in the literature but on study subjects *with* musculoskeletal problems. Thus a recent systematic review concluded that there was no effect of SM on the pressure pain threshold in people with musculoskeletal problems [18] A recent subsequent study, using the same sham as us with verification of blinding afterwards, also failed to identify an effect of SM on PPT in people *with* musculoskeletal problems [19].

However, there are other methodological issues than the sham that have to be taken into account in a successfully randomized controlled clinical trial.

### Methodological considerations concerning our study

Our previous systematic review [9] revealed that the most common methodological problems in this type of literature were the lack of blindness of statistician/statistical analysis, the failure to report losses and exclusions, and, importantly, the absence of blindness of the subjects. Our study avoided these errors. We ensured the blindness of the statistician by de-identifying the intervention groups (called A and B). Missing values and exclusion of data were reported and explained, but too few to change the results. Concerning the blindness of the subjects, a particular potential problem of our study was that our subjects consisted of chiropractic students (potential recruitment bias). They were likely to have previous knowledge of SM and to have a desire to show ‘positive’ results in favor of SM. To counteract this, we included only first year students. Apart from the use of post-study questionnaires, as explained above, we made sure that they were blind to i) the real purpose of the study, ii) to the interventions, iii) to the allocation of the groups, and iv) to the PPT readings.

Because the study subjects were all chiropractic students, even if this did not affect their ability to differentiate between intervention and sham, it is still possible that these results cannot be transposable to the general population. However, it could be argued that as the results were *not* in favor of SM, this recruitment factor would not have been significative in this study.

Other potential sources of error were also avoided by ensuring that the assessor was blinded to the type of intervention and that clinician and assessor were both experienced. Further, our PPT readings in the lumbar spine were found to be within the ‘normal’ range of values [20], whereas we could find no literature on ‘normal’ values in the thoracic spine.

### Other potential issues specific to the sham procedure

The validated sham procedure consisted of a pre-load tension with both hands on the medial part of the right scapula, followed by a ‘thrust’ to resemble as much as possible the real intervention. It could be argued that the ‘thrust’ movement on the scapula resembles too much a mobilization, with an active component capable of changing the pain perception in the subjects. However, the ‘thrust’ part was done on the scapula, i.e. outside the thoracic spine, and therefore could not be considered a form of spinal mobilization.

In our previous review [9] we theorized that a credible sham procedure should be acceptable both from a psychological point of view (*subjects found naïve, and blind*) and physiologically (*the sham resembling the active intervention*). Interestingly, we found in our previous review [9] that when the sham procedure was considered by us to be completely credible, the studies found positive results, with moderate general quality. In retrospect, this criterion may not have been sufficient, as it did not include an actual *validation* of the sham. In the light of our results, this previous definition seems not to have been sufficient.

### Other methodological considerations

Other experimental pain variables than PPT would perhaps have reacted differently to SM, such as pain induced by cold and heat, temporal summation (i.e. pain induced by repetitive irritations) and pain induced by irritating substances. However, a recent RCT on symptomatic subjects, testing lumbar SM using a validated sham included also temporal summation, with no effect [19]. This indicates that the lack of effect of SM is not primarily related to the type of test for pain.

### Interpretation of our findings

According to our study, SM has no specific effect on pain perception by pressure in the asymptomatic population. Recent literature indicates that this is also the case in the symptomatic population. The mechanisms of action of the SM on pain are therefore probably similar to those involved in a placebo maneuver, or at least the interpretation that the brain makes of these two interventions.

## Perspectives

- The validity of the sham intervention is essential in the study of the effects of manual therapies. It is necessary that the active intervention and the sham are as similar as possible in order to ensure the blindness of the subjects. In the same way, it is fundamental to always check the blindness of the subjects by the means of questionnaires.
- As suggested by one of the reviewers, in future studies the remote site should perhaps not only be non-dermatomal but also removed from the truncus, such as the elbow, wrist, knee or ankle.
- An important issue in relation to experimental studies of this type is to establish what level of improvement would be necessary before it corresponds to a clinically noticeable difference. This change should be above ‘the minimum change that would be greater than measurement error or chance’, calculated as between about 0.5 and 3.4 kg/cm ^2^ (20–50% change) for PPT [13, 21–23].
- Obviously, the effect size should be reported as well, but, as we have previously observed [11], this is often done in several and non-transparent ways, making real comparisons difficult.
- Another approach to grasp the ‘clinical’ validity of studies like this is to calculate the ‘Number Needed to Treat’ (NNT) [11]. For example, in our study we calculated how many subjects the study would need to obtain a statistically significant difference and found that more than 5000 subjects would be needed in each intervention group, assuming that the estimates remained unchanged.

## Conclusion

In conclusion, when compared to a valid sham procedure and with successfully blinded subjects, there is no regional or remote effect of spinal manipulation of the thoracic spine on the pressure pain threshold in a young pain-free population. Since our study was carefully designed and carried out and the results showed no relevant changes, we conclude that bigger and better studies are not to be recommended, at least not on asymptomatic people, manipulated in the thoracic spine measuring effect on the pressure pain threshold.

## Supplementary information

**Additional file 1.** Post-study questionnaire

**Additional file 2 **Descriptive data of the participants in the study (*n* = 50)

## Data Availability

The data used in the current study are available from the corresponding author on reasonable request.

## Abbreviations

NNT: Number needed to treat
PPT: Pressure pain threshold
RCT: Randomized controlled trial
SM: Spinal manipulation
