# Supplementary material for: What is the effect of spinal manipulation on the pressure pain threshold in young, asymptomatic subjects? A randomized placebo-controlled trial, with a cross-over design
Source: Chiropr Man Therap. 2020 Feb 7;28:6. doi: 10.1186/s12998-020-0296-1 (PMC7006124; doi:10.1186/s12998-020-0296-1)
Supplement: Supplementary file 1 — Additional file 1. Post-study questionnaire [file 12998_2020_296_MOESM1_ESM.docx]

**Additional file 1.** Post-study questionnaire

Subject ID:

Session ID:

**Post session questionnaire**

- I am definitely of the opinion that the intervention that I have received can modify the measured parameters
- I am rather of the opinion that the intervention that I have received can modify the measured parameters
- I do not know
- I am rather of the opinion that the intervention that I have received cannot modify the measured parameters
- I am definitely of the opinion that the intervention that I have received cannot modify the measured parameters
