# Supplementary material for: What is the effect of spinal manipulation on the pressure pain threshold in young, asymptomatic subjects? A randomized placebo-controlled trial, with a cross-over design
Source: Chiropr Man Therap. 2020 Feb 7;28:6. doi: 10.1186/s12998-020-0296-1 (PMC7006124; doi:10.1186/s12998-020-0296-1)
Supplement: Supplementary file 2 — Additional file 2 Descriptive data of the participants in the study (n = 50) [file 12998_2020_296_MOESM2_ESM.docx]

**Additional file 2.** Descriptive data of the participants in the study (n=50).

|  | **Mean (SD) or number ( %)** | | **Range** |
| --- | --- | --- | --- |
| **Age (years)** | 20 (+/- 3) |  | 18-37 |
| **Sex (female)** | 28 (56%) |  |  |
| **BMI (Kg/cm2)** |  |  |  |
| *> Underweight (<18,5)* | 1 |  |  |
| *> Normal (18,5-24,9)* | 38 |  |  |
| *> Overweight (25-29,9)* | 6 |  |  |
| *> Obese (>30,0)* | 0 |  |  |
| Missing data | 3 |  |  |

BMI : Body Mass Index
